# Use of Preoperative Apparent Diffusion Coefficients to Predict Brain Tumor Grade

**DOI:** 10.7759/cureus.2284

**Published:** 2018-03-07

**Authors:** Aneela Darbar, Muhammad Waqas, Syed Faaiz Enam, Shaikh D Mahmood

**Affiliations:** 1 Surgery, The Aga Khan University; 2 Biomedical Engineering, Duke University; 3 Medical College, The Aga Khan University

**Keywords:** brain tumors, apparent diffusion coefficient, magnetic resonance imaging, glioma grade

## Abstract

Introduction

The apparent diffusion coefficient (ADC) sequence is based on the diffusion properties of water molecules within tissues and correlates with tissue cellularity. ADC may have a role in predicting tumor grade for gliomas, and may in turn assist in identifying tumor biopsy sites. The purpose of this investigation was to assess the competence of preoperative ADC values in predicting tumor grades.

Methods

This was a retrospective investigation. We calculated the ADC values in the areas of greatest restriction in solid tumor components, and we recorded the pattern of contrast enhancement. Pathology reports masked to the imaging results were reviewed independently. We calculated the differences in the mean values of different tumor grades and high-grade and low-grade gliomas. A receiver operator curve (ROC) analysis assessed the predictive potential of ADC values for low-grade gliomas.

Results

Forty-eight cases of glioma were included in our study. We noted a statistically significant difference in the lowest mean ADC values for the tumor regions of Grade IV lesions (333.83 ± 295.47) compared with Grade I lesions (653.20 ± 145.07). On ROC analysis, we noted an area under the curve (AUC) of 0.80 for the lowest ADC value in the whole tumor region, which was a predictor of low-grade glioma with 95 % confidence interval (CI) of 0.675-0.926. The sensitivity of the lowest ADC value was 84.5% for high-grade lesions.

Conclusion

Given our findings that the means of the lowest ADC value are significantly different between low and high-grade gliomas with an AUC of 0.80 for ADC as a predictor of low-grade lesions and a sensitivity of 84.5% for high-grade lesions, ADC values contain some predictive properties of tumor grading. ADC values may be a valuable parameter in the assessment and treatment of tumors.

## Introduction

Diffusion magnetic resonance imaging (dMRI) of the human brain, first described in 1985 [1], has become an integral part of neuroimaging [1]. Apparent diffusion coefficient (ADC) is a modality based on the diffusion properties of water molecules within tissues [2]. The diffusion patterns of water molecules in the brain differ from true Brownian motion because water molecules bounce, cross, and interact with many tissue components, including cell membranes, fibers, and macromolecules [3]. Due to the heterogeneity of brain microarchitecture, normal and diseased areas of the brain have different ADCs [2]. Tissues with higher rates of diffusion have higher ADCs [4-5]. ADC, therefore, characterizes tissues quantitatively, and findings are validated in several studies [6-7]. For e.g., a study in 13 patients showed regional variations in cellularity, genetic expression, and ribonucleic acid (RNA) microarrays among regions with different ADCs [4].

A study in 16 patients with glioma showed that ADCs were lower in the brains of patients with glioblastoma multiforme (GBM) than Grade II astrocytoma and that ADC correlated with tumor cellularity [8]. ADC may also be predictive of tumor grade, although additional evidence is required [7].

GBMs are histopathologically evaluated by examining tissue samples obtained from regions of contrast enhancement (CE) on gadolinium-enhanced, T1-weighted images [9-12]. The sampling error of stereotactic surgery was found to be significant, leading to an underestimation of tumor grade, especially for high-grade lesions [13-14]. If ADC, a marker of cellularity, is predictive of tumor grade, then ADC may be useful in a more representative sampling of tumor tissues. This study, therefore, examined preoperative diffusion-weighted images to determine whether the ADC of gliomas correlates with the World Health Organization (WHO) grade.

## Materials and methods

This retrospective observational study was performed at the Aga Khan University Hospital (AKUH) in Karachi, Pakistan. The AKUH is a tertiary care hospital, accredited by the Joint Commission for International Accreditation (JCIA) and certified by the International Organization of Standardization (ISO), with over 43 specialties, including neurosurgery and radiology. The study period was two years, from January 2014 through December 2015, inclusive. Preoperative imaging and medical records were reviewed in February and March 2016. Data were reported according to Standards for the Reporting of Diagnostic Accuracy Studies (STRAD) [15], and the study was in complete compliance with the ethical human-subject standards of the World Medical Association Declaration of Helsinki: ethical principles for medical research involving human subjects [16].

Inclusion/exclusion criteria

All patients with a histopathological diagnosis of glioma presenting during the study period were included, irrespective of age, gender, and ethnicity. The study included gliomas of all grades and cell types (astrocytomas and oligodendrogliomas). Patients with indeterminate tumor grade or incomplete medical records and those who underwent preoperative radiology at a site outside the AKUH were excluded.

Magnetic resonance imaging (MRI) evaluation

Contrast enhancement was used as a reference test, whereas ADC was regarded as an index test. Gliomas were dichotomized as low (grades I and II) and high (grades III and IV) tumors according to the WHO classification. Two investigators assessed imaging data, and a third independently assessed histopathologic data. Both assessments were blinded to each other.

**Figure 1:**
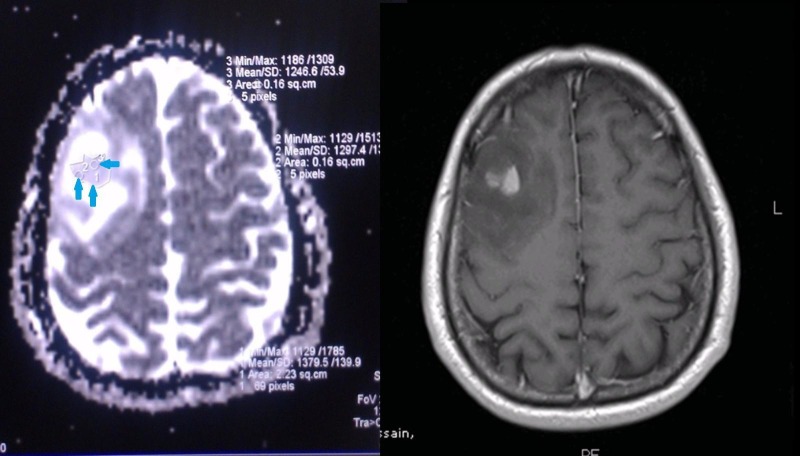
Contrast-enhancing Oligodendroglioma A contrast enhancing lesion in the right frontal lobe with significant mass effect. The darkest area on apparent diffusion coefficient (ADC) (left), however, had a mean value of 1246.6 (>800). The actual diagnosis is of grade II oligodendroglioma.

**Figure 2:**
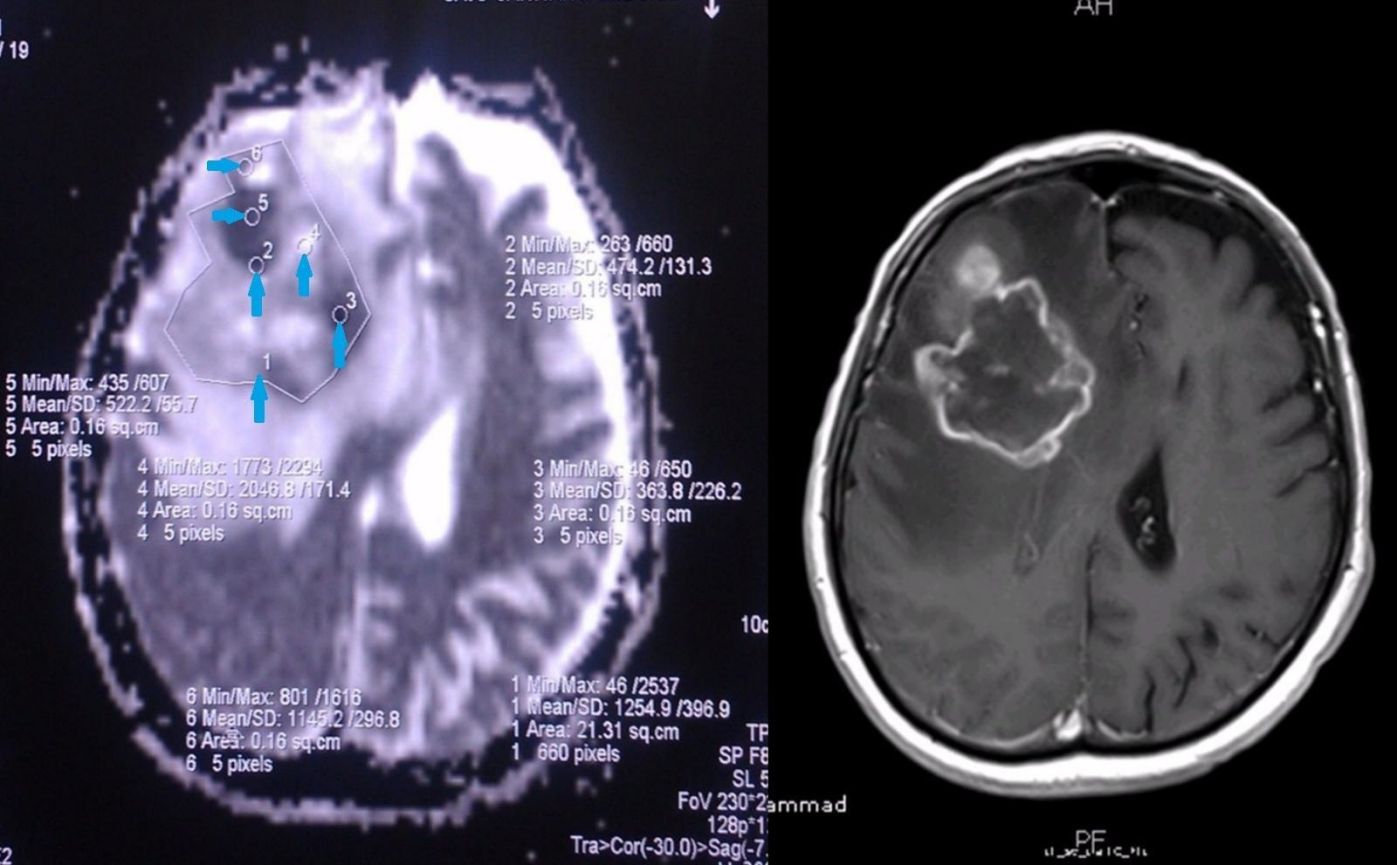
Contrast-enhancing Glioblastoma Multiforme (GBM) On apparent diffusion coefficient (ADC) sequence (left), the darkest area in the tumor region had a mean ADC value of 363.8 which according to the results of the receiver operator curve (ROC) represents high grade glioma. Contrast enhancement also favors the diagnosis. Histopathological diagnosis was GBM.

Diffusion imaging results were analyzed using the Siemens 1.5 Tesla Work Station (Siemens, Erlangen, Germany). The area of greatest diffusion restriction (lowest ADC) within the solid tumor component was identified while avoiding areas of peritumoral edema. Areas of visually obvious restricted diffusion within each tumor, defined as areas of bright signal intensity on b1000 images and a corresponding low ADC (i.e., a dark area on the ADC map), were identified. The first step in numeric ADC analysis was the examination of T1, T2, and post gadolinium sequences to identify and avoid areas likely to be cystic, hemorrhagic, calcific, necrotic, or peritumoral edema. The section with the lowest visual ADC within the remaining areas of the tumor was identified; a spherical region of interest (ROI) of 0.16 cm2 within this section was determined, and the ADC of this ROI was recorded. If the area of low ADC was large, the ROI was moved slightly within this area to determine the lowest ADC. The minimum ADC throughout the entire tumor and the mean ADC within the ROI of lowest ADC were calculated. A note was made of any difficulties in selecting the ROI, such as artifacts or small lesion size (Figures 1-2). Following the analysis of T1-weighted images with and without contrast, patterns of enhancement were described as heterogeneous, ring-like or diffuse.

Statistical analysis

All data were analyzed using SPSS v 17 (IBM, Chicago, Illinois, US). Frequencies of sample and tumor characteristics were calculated. Analysis of variance (ANOVA) was used to measure differences in mean ADCs of different tumor grades. Independent sample t-tests were used to compare mean ADCs in low and high-grade tumors. A P value <0.05 was considered statistically significant. Receiver operator curve (ROC) analyses were performed to determine whether ADCs were predictors of high-grade tumors, including the sensitivity and specificity of ADC as a predictor.

## Results

The study included 48 patients with glioma, 34 males (70.8%) and 14 females (29.2%), of mean age 40.59 ± 19.56 years. Fourteen patients were excluded because preoperative radiological imaging results were unavailable, and four were excluded because their histopathology results were inconclusive. Table 1 shows the baseline demographic and clinical characteristics of the patients in this study.

**Table 1:** Patient Demographics and Clinical Characteristics

Variables	Statistics
Demographics	Mean ± Std/ N (%)
Age (years)	40.56 ± 17.56
Gender	
Male	34 (70.8)
Female	14 (29.2)
Tumor characteristics	
WHO Tumor grade	
Low Grade	19 (39.6)
I	5 (10.4)
II	14 (29.2)
High Grade	29 (60.4)
III	11 (22.9)
IV	18 (37.5)
Tumor pathology	
Pilocytic Astrocytoma	5 (10.4)
Diffuse astrocytoma II	1(2.08)
Gemistocytic astrocytoma	1(2.08)
Oligodendroglioma II	12(25)
Anaplastic Astrocytoma	2 (4.16)
Anaplastic Oligodendroglioma	8 (16.67)
Anaplastic Oligoastrocytoma	1(2.08)
Glioblastoma Multiforme	14(29.16))
Gliosarcoma	4(8.3)
Enhancement Pattern	
Ring Enhancement	14(29.2)
Heterogeneous enhancement	27(56.3)
Diffuse	4(8.3)
No Enhancement	3(6.3)

The numbers and percentages of patients with grade I–IV tumors are shown in Table 2. The mean lowest ADC of the tumor region was significantly higher for grade I than for grade IV lesions (653.20 ± 145.07 vs. 333.83 ± 295.47, p = 0.001). Patients were dichotomized into those with high grade (III and IV) and low grade (I and II) gliomas. The mean lowest ADC of the tumor region was significantly higher for low grade than high-grade gliomas (678.73 ± 208.52 vs. 373.75 ± 257.06, p <0.001).

**Table 2:** Tumor Grades with ADC Values Std: standard deviation; ADC: apparent diffusion coefficient. *ANOVA test p value 0.001, **,*** t-test p value <0.001

Tumor Grade	Number (%)	ADC min ± Std	ADC lowest mean ± Std
Grade I	5 (10.4)	*653.20±145.07	1111.20±253.88
Grade II	14 (29.2)	687.85±231.06	1011.05±303.175
Grade III	11 (22.9)	439.09±170.41	755.12±191.59
Grade IV	18 (37.5)	333.83±295.47	727.90±233.59
Low Grade Gliomas	19 (39.6)	**678.73±208.52	1037.41±287.68
High Grade Gliomas	29(60.4)	***373.75±257.06	738.23 ±215.45

**Figure 3:**
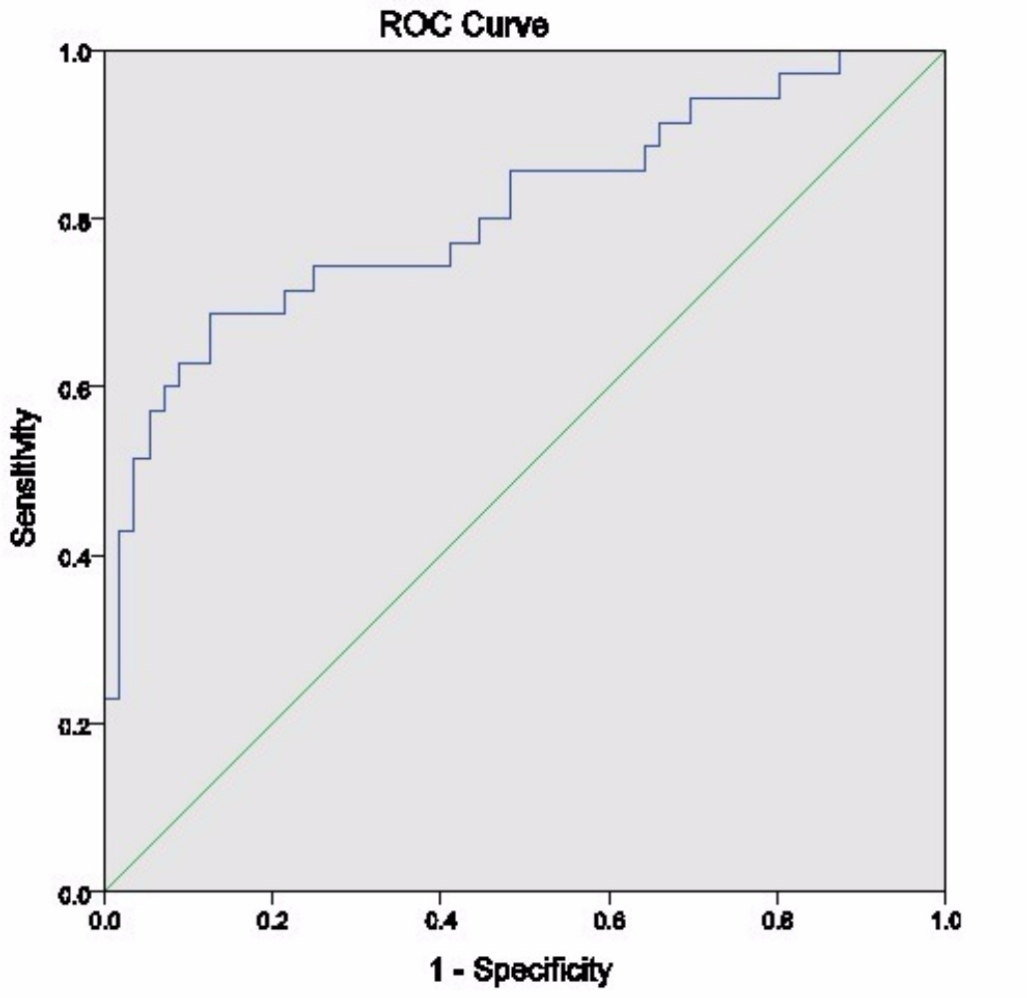
ROC Curve for Lowest Mean ADC as a Predictor of High Grade Tumors ADC: apparent diffusion coefficient; ROC: receiver operator curve.

ROC analysis showed that the area under the curve (AUC) for minimum ADC in the entire tumor as a predictor of low grade glioma was 0.80 (95 % confidence interval (CI) 0.675–0.926, p <0.001) and that the AUC for the mean lowest ADC sub-region was 0.80 (95% CI 0.66–0.937) (Figure 3). A cut off of 800 calculated from the ROC curve was used to estimate the sensitivity and specificity of ADC for high-grade gliomas (Table 3).

**Table 3:** Sensitivity and Specificity Analysis for ADC ADC: apparent diffusion coefficient.

	Tumor Grade		
ADC	High Grade	Low Grade	Total
Low <800	18	3	21
High >800	11	16	27
Total	29	19	48

Of the 48 tumors, three showed no enhancement on contrast, and one high-grade glioma showed diffuse enhancement on scanning. All estimates of diagnostic accuracy were conducted within 95% CIs.

## Discussion

The minimum ADC was significantly lower for high grade than for low-grade gliomas (373.75 ± 257.06 vs. 678.73 ± 208.52, p <0.001), and the minimum ADC for tumors had an AUC of 0.80 for predicting low-grade gliomas. Previous studies assessing the role of ADC in predicting tumor pathology [2, 8, 17] had several limitations, including small sample size and heterogeneous pathology [18]. The study with the largest sample size involved 275 patients, including subgroups with intra-axial lesions, and showed that ADC had good discriminatory value in predicting grades of astrocytomas [18]. ADCs have also been used to distinguish GBMs from lymphomas [19] and to reliably distinguish pilocytic astrocytomas from ependymomas and medulloblastomas in pediatric patients [20]. The lowest ADCs for grade I pilocytic astrocytoma in that study ranged from 800 to 2400, similar to our results [20]. However, that study did not include other types of glioma. To our knowledge, our study is the largest to date on patients with glioma and showed that ADC could distinguish among different tumor grades.

Although conventional imaging techniques of MRI and computed tomography (CT) have been widely used to diagnose brain tumors, they are not optimal for distinguishing between high grade and low-grade gliomas. Conventional MRI does not provide consistent information on factors that help determine tumor grade, including tumor microvascularity, micronecrosis, angiogenesis, and metabolism [21-22]. This inadequacy is also attributable to similarities in contrast enhancement, peri-lesional edema, necrosis and mass effect which can lead to low-grade gliomas being mistaken for higher grade ones and vice versa [21, 23]. According to Zhang et al., one-third of non-enhancing gliomas turn out to be high-grade gliomas, while on the other hand partial contrast enhancement is seen in around 20% of low-grade gliomas [21].

MRI in patients with GBM is markedly affected by tumor genetics and aggressive features, including cellularity and microvasculature [4]. Those findings were based on the ADC map of tumors, indicating that ADC maps can help in guiding the sampling of tumor regions, thereby improving the quality and representativeness of these samples. A prospective study showing the correlation between histologic features and ADCs can further validate these findings.

ADC is an appropriate imaging modality for prediction of tumor grades because of its capacity to accurately differentiate between low and high-grade tumors. Lower ADC values in high-grade gliomas are obtained due to increased cellularity of the tissue, while low-grade gliomas with less cellularity, and presumably a higher water content in interstitial spaces, result in increased ADC values [24].

Mean and minimum ADCs of the entire tumor, as well as the lowest mean ADC within a tumor, have been used to determine tumor pathology and grade [25]. Our study, which evaluated the minimum ADC of the entire tumor and the mean ADC of the subregion with greatest diffusion restriction, found that the latter had a higher AUC. Because most of the gliomas showed enhancement on contrast sequences, it may not discriminate among diagnostic grades.

In a recent study by Fawzy et al., diffusion MRI and ADC had a higher sensitivity, specificity, and accuracy than conventional MRI in glioma grading [26]. Based on currently available data, the pooled sensitivity and specificity of ADC maps calculated by Zhang et al. are 0.85 and 0.8, respectively, which further conveys the practicality of using ADC for differentiating between glioma grades [21].

Diffusion-weighted imaging and ADC, however, are not without pitfalls and limitations [27]. The images can be affected by artifacts, including motion and echo-planar imaging (EPI) artifacts and eddy currents. These limitations may be overcome by following standard protocols and are less common in the imaging of the brain than other organs of the body [27-28].

## Conclusions

Through this study, we aimed to analyze the potential of ADC in predicting tumor grade by examining diffusion-weighted images and correlating ADC of gliomas with the WHO grade. ADCs of tumor regions on preoperative MRI can discriminate between low and high-grade gliomas with an AUC of 0.80, and low-grade gliomas have significantly higher mean lowest ADCs than high-grade gliomas. ADCs greater than 800 are predictive of low-grade gliomas, with a sensitivity of 84.5%. We hope that these findings shed light on the possibility of a greater role of ADC in the representative sampling of tumor tissue, and believe that ADCs should be routinely evaluated when planning surgery for glioma.

## References

[1] Le Bihan D, Breton E, Lallemand D, Grenier P, Cabanis E, Laval-Jeantet M (1986). MR imaging of intravoxel incoherent motions: application to diffusion and perfusion in neurologic disorders. Radiology.

[2] Le Bihan D (2003). Looking into the functional architecture of the brain with diffusion MRI. Nat Rev Neurosci.

[3] Rowley HA, Grant PE, Roberts TP (1999). Diffusion MR imaging. Theory and applications. Neuroimaging Clin N Am.

[4] Barajas RF Jr, Rubenstein JL, Chang JS, Hwang J, Cha S (2010). Diffusion-weighted MR imaging derived apparent diffusion coefficient is predictive of clinical outcome in primary central nervous system lymphoma. Am J Neuroradiol.

[5] Stadnik TW, Chaskis C, Michotte A (2001). Diffusion-weighted MR imaging of intracerebral masses: comparison with conventional MR imaging and histologic findings. Am J Neuroradiol.

[6] Tien RD, Felsberg G, Friedman H, Brown M, MacFall J (1994). MR imaging of high-grade cerebral gliomas: value of diffusion-weighted echoplanar pulse sequences. Am J Roentgenol.

[7] Provenzale JM, Mukundan S, Barboriak DP (2006). Diffusion-weighted and perfusion MR imaging for brain tumor characterization and assessment of treatment response. Radiology.

[8] Kono K, Inoue Y, Nakayama K (2001). The role of diffusion-weighted imaging in patients with brain tumors. Am J Neuroradiol.

[9] Prayson RA, Agamanolis DP, Cohen ML (2000). Interobserver reproducibility among neuropathologists and surgical pathologists in fibrillary astrocytoma grading. J Neurol Sci.

[10] Coons SW, Johnson PC, Scheithauer BW, Yates AJ, Pearl DK (1997). Improving diagnostic accuracy and interobserver concordance in the classification and grading of primary gliomas. Cancer.

[11] Law M, Oh S, Johnson G (2006). Perfusion magnetic resonance imaging predicts patient outcome as an adjunct to histopathology: a second reference standard in the surgical and nonsurgical treatment of low-grade gliomas. Neurosurgery.

[12] Barajas RF Jr, Phillips JJ, Parvataneni R (2012). Regional variation in histopathologic features of tumor specimens from treatment-naive glioblastoma correlates with anatomic and physiologic MR imaging. Neuro Oncol.

[13] Jackson RJ, Fuller GN, Abi-Said D (2001). Limitations of stereotactic biopsy in the initial management of gliomas. Neuro Oncol.

[14] Revesz T, Scaravilli F, Coutinho L, Cockburn H, Sacares P, Thomas DG (1993). Reliability of histological diagnosis including grading in gliomas biopsied by image-guided stereotactic technique. Brain.

[15] Meyer GJ (2003). Guidelines for reporting information in studies of diagnostic test accuracy: the STARD initiative. J Pers Assess.

[16] (2001). World Medical Association Declaration of Helsinki. Ethical principles for medical research involving human subjects. Bull World Health Organ.

[17] Guo AC, Cummings TJ, Dash RC, Provenzale JM (2002). Lymphomas and high-grade astrocytomas: comparison of water diffusibility and histologic characteristics. Radiology.

[18] Yamasaki F, Kurisu K, Satoh K (2005). Apparent diffusion coefficient of human brain tumors at MR imaging. Radiology.

[19] Toh CH, Castillo M, Wong AM (2008). Primary cerebral lymphoma and glioblastoma multiforme: differences in diffusion characteristics evaluated with diffusion tensor imaging. Am J Neuroradiol.

[20] Jaremko JL, Jans L, Coleman LT, Ditchfield MR (2010). Value and limitations of diffusion-weighted imaging in grading and diagnosis of pediatric posterior fossa tumors. Am J Neuroradiol.

[21] Zhang L, Min Z, Tang M, Chen S, Lei X, Zhang X (2017). The utility of diffusion MRI with quantitative ADC measurements for differentiating high-grade from low-grade cerebral gliomas: evidence from a meta-analysis. J Neurol Sci.

[22] Weingart JD, McGirt MJ, Brem H (2010). High-grade astrocytoma/glioblastoma. Oncology of CNS Tumors.

[23] Ginsberg LE, Fuller GN, Hashmi M, Leeds NE, Schomer DF (1998). The significance of lack of MR contrast enhancement of supratentorial brain tumors in adults: histopathological evaluation of a series. World Neurosurg.

[24] Lee EJ, Lee SK, Agid R, Bae JM, Keller A, Terbrugge K (2008). Preoperative grading of presumptive low-grade astrocytomas on MR imaging: diagnostic value of minimum apparent diffusion coefficient. Am J Neuroradiol.

[25] Pierce TT, Provenzale JM (2014). Evaluation of apparent diffusion coefficient thresholds for diagnosis of medulloblastoma using diffusion-weighted imaging. Neuroradiol J.

[26] Fawzy FM, Almassry HN, Ismail AM (2016). Preoperative glioma grading by MR diffusion and MR spectroscopic imaging. The Egyptian Journal of Radiology and Nuclear Medicine.

[27] Le Bihan D, Poupon C, Amadon A, Lethimonnier F (2006). Artifacts and pitfalls in diffusion MRI. J Magn Reson Imaging.

[28] Provenzale JM, Engelter S, Petrella JR, Smith JS, MacFall JR (1999). Use of MR exponential diffusion-weighted images to eradicate T2 "shine-through" effect. Am J Roentgenol.

